# How do underage youth access e-cigarettes in settings with minimum age sales restriction laws? A scoping review

**DOI:** 10.1186/s12889-023-16755-9

**Published:** 2023-09-18

**Authors:** Anna Graham-DeMello, Janet Hoek, Jonathan Drew

**Affiliations:** 1 Department of Public Health, University of Otago, 23 Mein Street, Newtown, Wellington 6021 New Zealand; 2 Te Whatu Ora Southern, Health New Zealand, Dunedin Hospital, Dunedin, New Zealand

**Keywords:** e-cigarette, Vape, Youth, Underage access

## Abstract

**Background:**

Despite measures to reduce young people’s access to electronic cigarettes (ECs), or “vapes”, many countries have recorded rising youth vaping prevalence. We summarised studies documenting how underage youth in countries with minimum age sales restrictions (or where sales are banned) report accessing ECs, and outline research and policy implications.

**Methods:**

We undertook a focused literature search across multiple databases to identify relevant English-language studies reporting on primary research (quantitative and qualitative) and EC access sources among underage youth.

**Results:**

Social sourcing was the most prevalent EC access route, relative to commercial or other avenues; however, social sourcing dynamics (i.e., who is involved in supplying product and why) remain poorly understood, especially with regard to proxy purchasing. While less prevalent, in-person retail purchasing (mainly from vape shops) persists among this age group, and appears far more common than online purchasing.

**Conclusions:**

Further research examining how social supply routes operate, including interaction and power dynamics, is crucial to reducing youth vaping. Given widespread access via schools and during social activities and events, exploring how supply routes operate and evolve in these settings should be prioritized. Inadequate compliance with existing sales regulations suggest greater national and local policy enforcement, including fines and licence confiscation for selling to minors, is required at the retailer level.

**Supplementary Information:**

The online version contains supplementary material available at 10.1186/s12889-023-16755-9.

## Background

Early electronic cigarettes (ECs) simulated combustible cigarettes in appearance, but second and third generation devices became more powerful and more bespoke [1]. Fourth generation ‘pods’ and disposable devices differ from earlier models in their use of nicotine salts, a more palatable e-liquid that delivers higher nicotine concentrations without the harshness typical of freebase nicotine [2, 3]. EC marketing to youth has evolved rapidly across multiple media [4, 5], with a combination of aesthetically appealing, easily hidden, and powerfully addictive devices contributing to rising EC use among young people [6, 7].

While views on ECs’ role in supporting smoking cessation vary [8–10], most researchers agree that young people who do not smoke face possible harm if they begin using ECs [11]. Physical harms include respiratory risks and the as yet unknown impact of sustained EC use; psychological risks include the burden dependence imposes, which may lead to anxiety, shame, and financial stress [12, 13]. Further, although causal links are disputed [14, 15], researchers have found associations between EC use and subsequent smoking [15–21], leading some to conclude that youth who use ECs are three times more likely to begin using smoked tobacco products than those who do not use vaping products [16, 17].

Policy makers in some countries have attempted to support movement to ECs among people who smoke and cannot quit, while preventing uptake among young people who have never smoked [22–25]. Canada, England, and Aotearoa New Zealand (NZ) have set a minimum legal sales age of 18 years [26], while Tobacco 21 legislation in the US increased the minimum legal sales age in that country to 21 [27, 28]. Australia has banned consumer sales of nicotine-containing ECs and requires people to obtain prescriptions before they may legally access these products [29, 30]. Many countries require on-pack warnings and have prohibited promotions using traditional advertising media [29], and some have limited access to or banned the fruit and confectionary options popular among young people [29].

Yet, despite these policy initiatives, EC use among youth has continued to rise in Canada, the UK, Australia, NZ and elsewhere [31–37]. While the 2019–20 National Youth Tobacco Survey suggested EC use had declined among US youth during that period, overall prevalence remained high and trends toward declining use are not yet established [38].

Efforts to reduce ECs’ appeal and availability to young people have thus not proved successful and evidence underage youth access ECs raises important questions about the supply routes they use. We therefore undertook a scoping review with two objectives: to summarise evidence of how underage youth from countries with established minimum age sales restrictions or bans report accessing ECs, and to analyse how that knowledge could inform future research and policy [39]. We compiled and synthesised data on supply sources, including social (via friends, family or other contacts), commercial (retail self-purchasing, whether in-person or online), and other (more obscure or undefined) access routes.

## Methods

### Search strategy

In September 2022 ADM and a University of Otago subject librarian conducted a search of peer reviewed literature across four databases. We restricted study language to English and dates from 2015 (the year in which US youth vaping appears to have peaked and before which age national restrictions had not yet been implemented) [40]. Additional File 1 outlines the search strategy, including limits and search terms applied within each database.

### Study selection

ADM and JAH screened titles independently, (*n* = 1,870) then met to reach consensus and develop an agreed list for further review. ADM and JD then screened abstracts independently (*n* = 215), meeting to reach consensus. JAH further reviewed the agreed list. We included studies if they reported on primary research and EC access routes among those < 18 years of age (or those < 19 or < 21 years of age, depending on minimum age sales laws in the country of interest). We excluded studies that focused exclusively on: access to other tobacco products or cannabis; knowledge, attitudes, prevalence, or correlates of EC use; advertising or marketing; biomedical findings, or health outcomes. We also excluded studies conducted prior to implementation of EC minimum age sales laws. Additional File 1 presents inclusion and exclusion criteria, and details of study numbers at each screening phase. Additional File 2 provides an overview of these laws across the four countries represented in this review.

### Data extraction and synthesis

ADM created a Microsoft Excel spreadsheet to facilitate extraction of descriptive data (variables included study citation, setting, study type, objectives, methods, eligibility criteria, participant demographics, data collection dates, EC access parameters reported, ethical considerations, conflicts of interest, and funding sources). We used this spreadsheet to summarise study characteristics and identify methodological similarities and differences within included studies. ADM and JD extracted and tabulated quantitative data from surveys into a separate spreadsheet, detailing the number (and proportion) of respondents who reported access via various sources. Given variability in response categories across included surveys, ADM and JD identified matching EC access route descriptors to facilitate data synthesis.

## Results

### Overview of included studies

**Fig. 1:**
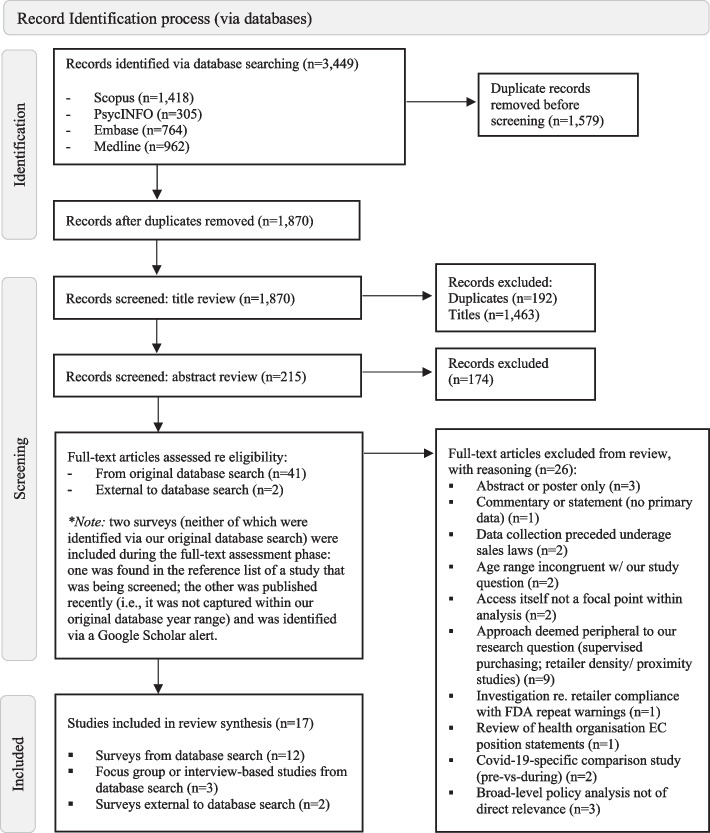
Search flow, including record identification, screening, and full-text assessment stages

We included 17 studies in this scoping review; Fig. 1 presents the search flowchart from record identification to full-text assessment. Additional File 3 outlines characteristics and variables from included studies.

Most included studies (*n* = 14) were conducted in the United States (US) [41–54]; one was conducted in Canada [55]; one in Australia [34], and one was international (US, Canada, England) [56]. All were published in peer-reviewed journal articles; most (*n* = 14) were surveys and half of these reported data from a nationally representative sample [43–47, 50, 55]. Two studies reported on focus-groups [52, 54] and one on in-depth interviews [53]. The median start date for data collection was 2017 [34, 41–56].

For analyses relevant to underage EC access, most surveys (*n* = 11) included data from more than 1,000 participants [43–51, 55, 56]; across all surveys (*n* = 14), the mean number of participants was 2,198 (range = 7,979) [34, 41–51, 55, 56]. Focus-group and interview-based studies included between 29 and 61 participants [52–54]. Sample members identified as male or female and typically ranged from age 13 to 18 years (*n* = 8).

Most studies (*n* = 12) included participants classified as ‘current’ or ‘past 30-day’ EC users (all current users had vaped in the past 30 days) [42–47, 50–53, 55, 56], though fewer (*n* = 5) included underage ever-users [34, 41, 48, 49, 54]. A single study reported receiving industry funding (from JUUL Labs, Inc.; the study explored access to JUUL EC products only) [44].

### EC access modes: social, commercial, other

Most included surveys (*n* = 11) found social sources were the most prevalent means underage youth used to access ECs, relative to commercial or other (mainly undefined) sources [34, 41, 42, 44–47, 49, 50, 55, 56]. A single survey found commercial sourcing was most prevalent [48].

**Fig. 2:**
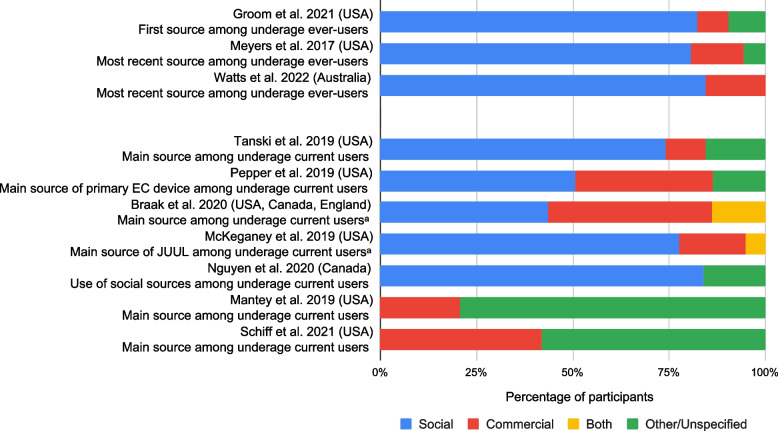
Prevalence of Key EC Access Modes Among Ever- and Current Users. ^a^ Study provided data re. exclusive sourcing from commercial vs other/unspecified avenues among current EC users (i.e., as a proportion of 100%), thus allowing for graphical presentation along with studies that allowed a single response option to questions re. EC access source. NB: First or most recent source among EC ever-users was reported in three studies, while main source among current EC users reported in seven; the two study groupings are separated by an additional space within the figure. NB: Response categories contained within ‘social’ sourcing include: friend, family, neighbour, or another contact (not a friend or relative) who either shared with, lent to, gave, or sold to the person, or bought on their behalf; response sub-categories contained within ‘commercial’ sourcing include: having self-purchased in-person at a vape shop or any other type of retailer, online, or at a flea market; response sub-categories contained within ‘other/unspecified’ sourcing include: stealing or taking, or not providing specific details re. sourcing (e.g., ‘some other way’ or ‘don’t remember’)

Heterogeneity in question framing within included studies affected how we present our findings. Eight surveys allowed a single response to questions examining EC access source (i.e., prevalence totalled 100%), thus allowing us to match and combine responses categories into three access modes: ‘social’, ‘commercial’, and ‘other/ unspecified’ [34, 41, 43, 46, 47, 49, 51, 55]. Two surveys provided data regarding exclusive sourcing from ‘social’ vs ‘commercial’ vs ‘both’ avenues [44, 56]. Figure 2 presents these 10 studies.

Social sourcing was most prevalent in eight of 10 studies, ranging from 43%-85% (weighted mean = 75%) [34, 41, 44, 46, 47, 49, 55, 56]. In five of these eight studies, social was followed by commercial sourcing, ranging from 14%-41% (weighted mean = 30%) [34, 41, 44, 46, 56]; in three, social was followed by other/unspecified sourcing, ranging from 10%-16% (weighted mean = 15%) [47, 49, 55]. Two studies did not describe access outside commercial purchasing [43, 51], thus we could only assess commercial vs other/unspecified sources (21% vs 79% and 42% vs 58%, respectively).

Among the six surveys allowing respondents to cite multiple sources, four did not provide additional data on exclusive sourcing (i.e., graphical presentation as in Fig. 2 was not feasible). Surveys reporting on main sources among current EC users found a social source was the single most prevalent response category, ranging from 43%-59% (weighted mean = 50%) [42, 44, 45, 50, 56]. One survey reporting on main sources among EC ever-users found a commercial source the single most prevalent category, ranging from 41%-61% across five individual EC device types probed (JUUL, pod vape, Cigalike, Box-Mod, and vape pen), while social sources were the second and third most prevalent categories across all five device types (9%-22% and 20%-28%, respectively) [48].

### Social sourcing sub-analysis

#### Friend

All six surveys including ‘Friend’ as a category found this option was the single most prevalent source (social, commercial, or otherwise). Prevalence ranged from 52%-60% (weighted mean = 59%) across those reporting on either first or most recent source among EC ever-users [34, 41, 49], and from 47%-55% (weighted mean = 52%) across those reporting on main source among current EC users [42, 45, 50].

Five surveys did not include ‘Friend’ as a response category but included one or more of the following options: ‘someone gave’, ‘someone offered’, or ‘I asked someone to give me some’, which imply social sourcing [44, 46–48, 56]. A single study examined borrowing and sharing behaviour and reported on prevalence and friend-specific involvement [46]. Regardless of high device ownership levels, 73% of survey respondents reported borrowing someone else’s device in the past 30 days (nearly 1 in 5 borrowed frequently in that period); friends were the primary borrowing source (81%), followed by siblings (9.5%); less than five percent borrowed from parents/adult relatives, co-workers, or others. Sharing devices was also common, with 37% reporting they often or very often shared a device [46].

#### Family

Seven surveys included ‘Family’ (whether described as such, or as ‘Parent/Legal Guardian’ or ‘Sibling’) as a response option when examining EC access sources [34, 41, 42, 45, 46, 49, 50]. All seven found family was a much less prevalent source relative to friends, estimates reported from 9%-16% (weighted mean = 15%) of EC ever-users reporting on either their first or most recent source [34, 41, 49], and from 5%-28% (weighted mean = 11%) of current EC users reporting on their main source [42, 45, 46, 50].

#### Social purchasing

Six surveys examined social purchasing (i.e., asking someone to purchase or having purchased from someone) as a distinct source option. Response prevalence for *‘I got someone else to buy on my behalf’* ranged from 5%-23% (weighted mean = 15%) across those reporting on either main or most recent source among EC ever-users [34, 48]; and from 13%-43% (weighted mean = 24%) across those reporting on main source among current EC users [44, 46, 47, 56]. Response prevalence for *‘I bought from another person’* ranged from 9%-16% (weighted mean = 12%) across studies reporting on main source among current EC users [44, 46, 56].

While most reviewed studies provided no further detail about who made social purchases or the importance of these sources, two examined purchases from friends and reported prevalence (as a proportion of social purchases only) of 4% and 75% [34, 44]. One study found prevalence of purchases from family (as a proportion of social purchases) was one percent [44].

#### Social sourcing insights from focus groups and interviews

All three included qualitative studies found underage youth often obtained their first EC via their peers [52–54], with initial use commonly occurring with friends or siblings at home, at school, or in another public space (e.g., a park) [52, 53]. Device sharing usually occurred on school grounds [52–54] or in other social settings where there was little oversight [53, 54]. Older friends or siblings of legal purchase age played an important role in supplying younger underage youth [52, 54].

### Commercial purchasing sub-analysis

**Fig. 3:**
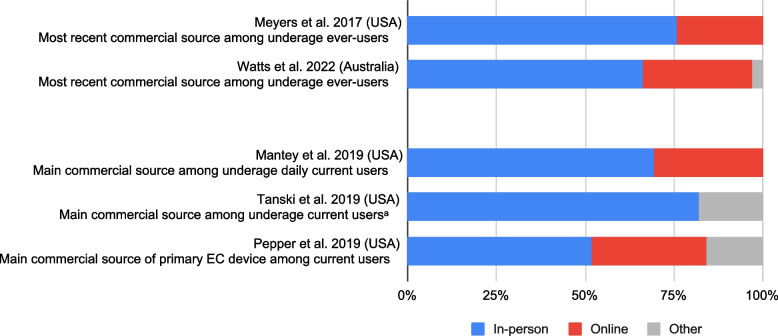
Prevalence of EC Commercial Purchasing Modes Among Ever- and Current Users. ^a^ Too few respondents purchased ECs online to report a proportion that would be statistically reliable. NB: Most recent source among EC ever-users was reported in two studies, and main source among current EC users was reported in three; the two study groupings are separated by an additional space within the figure. NB: Response categories contained within ‘in-person retail’ include: vape shop/store, smoke shop, tobacconist, convenience store, gas or petrol station, liquor store, drug store, mall kiosk; response categories contained within ‘online’ sourcing include: internet, website, or via a social media site; response categories contained within ‘other’ sourcing include: other location, ‘purchased, but not from retail store (e.g., market)'

Five surveys allowing only a single response option to questions assessing EC access source enabled us to match and combine response categories into three commercial purchase categories: ‘in-person’, ‘online’, and ‘other’. Figure 3 presents these studies. In-person retail was the most prevalent source, ranging from 52%-82% (weighted mean = 62%) [34, 41, 43, 46, 47]. In four of these five studies, in-person was followed by online sourcing, ranging from 24%-32% (weighted mean = 31%) [34, 41, 43, 46]; in one, in-person was followed by ‘other’ sourcing (18%) [47].

#### Vape shop

Eight surveys included ‘vape shop’ as a response category for EC access questions. Among those reporting on either main or most recent source among EC ever-users, response prevalence ranged from 4%-45% (weighted mean = 27%) [34, 48], while those reporting on current EC users found response prevalence ranged from 4%-28% (weighted mean = 14%) [42, 44–46, 50, 51].

#### Convenience store/ gas station

Five surveys reporting on main source(s) among current EC users, included ‘gas station or convenience store’ as a response category; prevalence ranged from 6%-22% (weighted mean = 12%) [44–47, 50].

#### Smoke shop/ tobacconist

Six surveys included either ‘smoke shop’ or ‘tobacconist’ as a response category; among those reporting on either main or most recent source among EC ever-users, response prevalence ranged from 2%-16% (weighted mean = 6%) [34, 41, 48]. Those reporting on main source(s) among current EC users reported response prevalence ranging from 5%-18% (weighted mean = 7%) [46, 47, 51].

#### Online

Eleven surveys included ‘online’ as a response option; among those reporting on either main or most recent source among EC ever-users, prevalence ranged from 5%-37% (weighted mean = 28%) [34, 41, 48]. Those reporting on main source(s) among current EC users reported prevalence ranging from 3%-16% (weighted mean = 8%) [34, 42–46, 50, 56].

#### Commercial purchasing insights from focus groups and interviews

In two qualitative studies, underage self-purchasers commonly bought from in-person retailers who either did not verify their age or accepted a fake ID. One study found that larger shops or chain stores (e.g., 7-Eleven in the US) were more likely than small local shops to enforce age verification [53].

## Discussion

Our scoping review summarises methodologically diverse studies that explored sources underage youth use to access ECs. Most included surveys found social sources to be the most prevalent EC access route among underage youth [34, 41, 42, 44–47, 49, 50, 55, 56], compared with commercial or other avenues. Only one survey found commercial sourcing was the most prevalent EC access source [48]. However, this study was limited to EC device owners so would not have detected borrowing and sharing practices among non-owners; it may thus have under-estimated actual social sourcing among underage youth.

Survey data showed friends were the most prevalent social source; [34, 41, 42, 45, 49, 50] only one survey reported on device sharing [46], which recent research suggests merits further investigation [34]. Though proxy purchasing constituted an important form of social sourcing [34, 44, 46–48, 56], most surveys did not identify the parties involved in these transactions; the dynamics involved therefore remain poorly understood. Two surveys reported on purchasing via friends [34, 44], though varying prevalence made it difficult to offer clear interpretations. Data from focus groups and interviews also highlighted friends’ roles in initial and ongoing EC use, particularly at schools [52–54].

Survey data on commercial sources, including in-person purchases, revealed these were a more prevalent source than online purchases [34, 41, 43, 46, 47]; with vape shops the most common location for in-person purchases [34, 42, 44–46, 48, 50, 51]. Focus group and interview data revealed underage youth purchase from retailers known to have lax age verification processes [52, 53].

### Implications for research and policy

Existing minimum age sales laws and sales bans appear not to have adequately prevented youth from accessing ECs in several countries included in this review [34, 55, 56]. Further evaluation of US T21 legislation is required to assess whether a trend in declining prevalence is evident and to explore the impact this measure has had on social sourcing [42, 47, 51, 56, 57]. Given social sourcing commonly occurs in schools and other public spaces [52–54], future research should probe how supply routes evolve and operate within these settings. We support calls to explore sharing and borrowing behaviour, as well as device gifting and proxy purchases, in greater depth [42, 46, 49], Understanding interaction dynamics, and the parties and power structures involved, could inform policies to effectively disrupt social supply channels [44]. Probing factors that motivate EC experimentation and early use among underage youth (considering sex-specific differences, if and where appropriate) could support targeted preventive policies [42, 55, 57], including stronger and well-enforced marketing curbs [42, 56] flavour restrictions [42, 55, 58–60], and evidence-based youth prevention campaigns.

While social supply appears more prevalent than commercial supply, underage youth report using commercial routes when self-purchasing. Inadequate compliance with existing sales regulations suggest greater national and local policy enforcement, including fines and licence confiscation for selling to minors, is required [49, 51, 55, 56, 61–63].

To address the research gaps identified above and improve comparability between studies, we suggest developing a standardised approach, including common questions. Rigorous evaluation of policies aimed at limiting youth EC access would provide a robust basis for future policy development [41, 62]. For instance, better knowledge of how national legislation (e.g., T21 measures), flavour restrictions, and public education campaigns have contributed to decreases in EC use among US youth could identify opportunities to strengthen existing measures [50].

### Strengths and limitations

We analysed data sourced from diverse studies representing around 30,000 participants, developed the first review analysing how underage youth access EC products, and offer new insights regarding EC use among underage youth.

Our scoping review has limitations as the published literature has a limited geographic scope; most studies were conducted in the US, and although Canada, England, and Australia are also represented, the findings may not generalise to other settings. The survey questions reviewed varied in their wording (e.g., probing of first, most recent, or main EC source), structure (e.g., single vs multiple responses options), and response categories (i.e., variable disaggregation of social, commercial, and other sources), making data synthesis challenging. Because included studies were cross-sectional, we could not describe access trends or source preferences over time. Finally, as circumstances surrounding EC product development and regulation continue to change rapidly [64], and because we did not critically appraise the included articles, we have exercised caution in outlining policy implications and instead identify research that could inform future policy [65].

## Conclusions

Underage youth reported on in the studies we reviewed typically used social sources to access ECs. Explicating how social supply routes operate is crucial to reducing youth vaping. Widespread access to ECs at schools and social events makes examining interaction dynamics and supply route operation in these settings an urgent research priority, alongside effective sales and marketing controls.

### Supplementary Information

**Additional file 1.** Detailed search strategy, screening stages, and discard reasoning.

**Additional file 2.** Minimum age sales restrictions by Country and region of interest.

**Additional file 3.** Detailed characteristics of included studies.

## Data Availability

Data generated during this study are included in this published article; all data included in our analysis (from previously published peer-reviewed research articles) are available from the corresponding author on reasonable request.
