# Supplementary material for: How do underage youth access e-cigarettes in settings with minimum age sales restriction laws? A scoping review
Source: BMC Public Health. 2023 Sep 18;23:1809. doi: 10.1186/s12889-023-16755-9 (PMC10506222; doi:10.1186/s12889-023-16755-9)
Supplement: Supplementary file 1 — Additional file 1. Detailed search strategy, screening stages, and discard reasoning. [file 12889_2023_16755_MOESM1_ESM.docx]

Additional File 1: Detailed Search Strategy, Screening Stages, and Discard Reasoning

Table S1(a): Search Strategy- Databases, Search Terms, and Results

| **Database** | **Search Terms** | **Results** |
| --- | --- | --- |
| Scopus  *Year: 2015-present*; English language only;*  *(further limited to articles, reviews and short surveys)* | youth OR "young person" OR "young people" OR "young adult*" OR teen* OR adolescen* OR underage OR "under-age" OR minor*  AND  vape OR vapes OR vaping OR "vape pen*" OR "vape stick*" OR "electronic cigarette*" OR “e cigarette*” OR “e-cigarette*” OR "e cig*" OR "e-cig*" OR "electronic nicotine" OR “juul”  AND  supply* OR purchas* OR buy* OR access* OR sourc* OR acqui* OR obtain* OR get* OR procur* OR receiv* OR retail* OR online OR “black market”, “black-market” OR underground | 1,418 |
| PscyINFO  *Year: 2015-present; English language only;*  *(further limited by age group: adolescents 13-17 years and young adults 18-29 years)* | Same keyword search  + Subject Headings:   - ‘Electronic Cigarettes’ (covers c-cigarette, e-cig, electronic nicotine delivery system, and personal vaporizer); - ‘Retailing’ (also covers ‘store’) | 305 |
| Embase  *Year: 2015-present; English language only;*  *(further limited by age group: adolescents 13-17 years)* | Same keyword search  + Subject Headings:   - ‘Juvenile’ (covers youth and adolescent); - ‘Minor (person)’; - ‘Vaping’ (covers e-cigarette smoking, electronic cigarette smoking); - ‘Electronic Cigarette’ (covers electronic cigarette/s, e cigarette/s, electronic nicotine delivery system/s) - ‘Retail Outlet’ (covers consumer outlet/shop, retail store/market/ shop/ facility, street market, public market) - ‘Convenience Store’ (convenience shop, corner shop/store) | 764 |
| Medline  *Year: 2015-present; English language only;*  *(further limited by age group: adolescents 13-18 years and young adults 19-24 y)* | Same keyword search  + Subject Headings:   - Adolescent; - Young Adult; - ‘Vaping’; - ‘Electronic Nicotine Delivery Systems’ | 962 |
| Total references: 3,449  Duplicates across all searches: 2,559 (2, 3, or 4 duplicates per identified reference)  Duplicate records removed before screening: 1,579 | | |

**NB: ‘Present’ signifies September 1st, 2022*

Table S1(b): Inclusion and Exclusion Criteria; Breakdown of Screening Stages

| **Inclusion/ Exclusion Criteria**  Inclusion criteria   - Includes adolescents (age range 13-17), or very young adults in USA (or elsewhere) where minimum age sales restrictions for ECs now apply to those up to 21 years of age (i.e., ages 18-21) - Focuses at least in part on EC (electronic cigarette) availability, access, or supply routes - Empirical study (qualitative or quantitative) of youth - Review, meta-analysis   Exclusion criteria   - Focus on adult EC access or supply and does not include adolescents or very young adults - Focus on other policies (e.g., flavours, gateway hypothesis, advertising and promotion policy) and does not include access or supply - Focus on smoked tobacco or cannabis not EC - Focus on health conditions (e.g., EVALI, asthma, Covid-19) - Biomedical research (e.g., animal testing, lab-based studies) - Focus on knowledge, attitudes or behaviour (e.g., prevalence) without considering access or supply - Focus on prevalence and correlates without considering access or supply - Focus on advertising impacts/outcomes without considering access or supply - Focus on youth vaping behaviour during Covid-19 pandemic - Article is a commentary, letter, or statement (no primary/empirical data presented) - Research was conducted before implementation of underage sales restrictions in country or region of interest - Not in English language |
| --- |
| **Title Screening**   - Total references for title screening: 1,870 - Duplicates identified/removed via title screening itself (not identified by Endnote): 192 - Total after duplicates removed: 1,678 - Total number of records removed via title review (did not meet inclusion criteria): 1,463 - Total records for abstract screening: 215 |
| **Abstract Screening**   - Total records for abstract screening: 215 - Both reviewers agreed to exclude: 174 - Both reviewers agreed to include: 33 - Reviewers disagreed and then met to reach consensus: 8 - Studies selected for full-text assessment (original database search): 41   **Note: two additional surveys also selected externally to database search (one identified within an included study and another published post-lit. search)*   - Total records for full-text assessment: 43 |
| **Full-text Assessment**   - Total records for full-text assessment: 43 - Total records that did not meet criteria upon full-text review, with reasoning: 26 - Studies included in narrative review (n=17): - Surveys: 14 (12 from original database search; 2 external to database search); - Focus group or interview-based studies: 3 |

Table S1(c): Studies excluded at full-text review stage (categorisation and reason for discard)

| **Categorisation** | **Discard Reasoning** |
| --- | --- |
| Survey, in-person (n=1)  Survey, online (n=2) | Not a published study (only abstract or poster available) |
| Survey, in-person (n=2) | Data collection precedes implementation of underage sales law in region or country of interest |
| Interviews, telephone (n=1)  Interviews, in-person (n=1) | Age range does not align with study question, or no separate analysis conducted for underage youth |
| Survey, in-person (n=1)  Systematic review (n=1) | Access modes/routes not a focal point within analysis |
| Online purchasing study (n=2)  In-store purchasing study (n=2)  Website analysis (n=1) | Method centers on observation of minors making supervised EC purchase attempts from vendors, or on website content analysis; approach seen as peripheral to research question |
| Retailer density/proximity study (n=4) | Method centers on compiling density and/or proximity data for EC retailers within a certain distance of schools or neighbourhoods; approach peripheral to research question |
| Review article (n=1)  Retailer compliance investigation (n=1) | Method centers on reviewing health organisation position statements re. ECs, or on compliance with FDA directives to an organisation; not directly relevant to research question |
| Survey, online (n=2) | Method centers on comparing EC use during Covid-19 pandemic to pre-pandemic, or on access in the context of ‘stay at home’ orders; peripheral to research question |
| Policy commentary (n=1)  Policy overview (n=3) | Commentary or statement (no primary data), or broad-level policy paper not directly relevant to research question |
