# Supplementary material for: How do underage youth access e-cigarettes in settings with minimum age sales restriction laws? A scoping review
Source: BMC Public Health. 2023 Sep 18;23:1809. doi: 10.1186/s12889-023-16755-9 (PMC10506222; doi:10.1186/s12889-023-16755-9)
Supplement: Supplementary file 2 — Additional file 2. Minimum age sales restrictions by Country and region of interest. [file 12889_2023_16755_MOESM2_ESM.docx]

Additional File 2: Minimum Age Sales Restrictions by Country and Region of Interest

Table S2: Implementation Dates for EC Minimum Age Sales Laws

| United States (State-level) | |
| --- | --- |
| *California*  *North Carolina*  *Florida*  *Connecticut* | - September 2010: State law prohibits sales to minors (<18);[1] - June 2016: State T21 law raises minimum legal purchase age for all tobacco products, including ECs, to 21years;[2,3] - August 2013: State law prohibits sales to minors (<18);[4] - July 2014: State law prohibits sales to minors (<18);[1] - October 2014: State law prohibits sales to minors (<18);[1]   Note: 47 US states and 2 territories already prohibited EC sales to those <18 as of 2014, though limited state resources call into question whether enforcement was robust; FDA did not yet regulate ECs at federal level)[5] |
| Unites States (Nation-wide) | |
| *FDA Regulation*  *FDA Regulation* | - August 2016: FDA prohibits EC sales to minors <18 at federal leve[6] - December 2019: FDA raises federal minimum legal purchase age for tobacco products, including ECs, from 18 to 21 years[7] |
| Canada (Provincial-level) | |
| *Nova Scotia*  *New Brunswick*  *PEI*  *Quebec*  *Ontario*  *Newfoundland/ Labrador*  *British Columbia*  *Manitoba*  *Alberta*  *Saskatchewan* | Note: Provincial bans on ECs to youth <18 or <19 (depending on province) were first implemented between 2015 and 2017 (as outlined below);   - May 2015: Province prohibits EC sales to minors <19;[8] - July 2015: Province prohibits EC sales to minors <19;[8] - October 2015: Province prohibits EC sales to minors <19;[8] - November 2015: Province prohibits EC sales to minors <18;[8] - January 2016: Province prohibits EC sales to minors <19;[8] - June 2016: Province prohibits EC sales to minors <19;[8] - September 2016: Province prohibits EC sales to minors <19;[8] - October 2017: Province prohibits EC sales to minors <18;[8] - No ban: N/A;[8] - No ban: N/A; [8] |
| Canada (Nation-wide) | |
| *Federal Regulation* | - May 2018: Nation-wide, sales prohibited to minors <18, though legal purchase age in certain provinces/territories may be higher (i.e., 19 or 21);[9] |
| England (Nation-wide) | |
| *Federal Regulation* | - March 2015: Nation-wide, sale of nicotine products (including ECs) prohibited to minors <18 via Children and Families Act;[10] |
| Australia (State-level) | |
| *New South Wales (NSW)* | - December 2015: State law prohibits sale of vape products/accessories to minors <18, via amendments to NSW Public Health (Tobacco) Act 2008;[11] |
| Australia (Nation-wide) | |
| *Federal Regulation* | - October 2021: Nation-wide, via nicotine scheduling changes, all Australians require a prescription to legally access nicotine-containing EC products for any purpose.[12] |

REFERENCES

1. Centers for Disease Control and Prevention (CDC). State Laws Prohibiting Sales to Minors and Indoor Use of Electronic Nicotine Delivery Systems — United States, November 2014. Accessed December 12, 2022. https://www.cdc.gov/mmwr/preview/mmwrhtml/mm6349a1.htm

2. Schiff S, Liu F, Cruz TB, et al. E-cigarette and cigarette purchasing among young adults before and after implementation of California’s tobacco 21 policy. *Tobacco Control*. 2021;30(2):206-211. doi:10.1136/tobaccocontrol-2019-055417

3. Bonnie RJ, Stratton K, Kwan LY, eds. *Public Health Implications of Raising the Minimum Age of Legal Access to Tobacco Products*. National Academies Press (US); 2015. Accessed December 12, 2022. http://www.ncbi.nlm.nih.gov/books/NBK310412/

4. Moore J. Regulating Electronic Cigarettes in North Carolina, Part 1: Federal and State Regulation. Published July 31, 2014. Accessed December 12, 2022. https://canons.sog.unc.edu/2014/07/regulating-electronic-cigarettes-in-north-carolina-part-1-federal-and-state-regulation/

5. Tanski S, Emond J, Stanton C, et al. Youth Access to Tobacco Products in the United States: Findings from Wave 1 (2013-2014) of the Population Assessment of Tobacco and Health Study. *Nicotine and Tobacco Research*. 2019;21(12):1695-1699. doi:10.1093/ntr/nty238

6. US Food and Drug Administration (FDA). How FDA is Regulating E-Cigarettes. Published August 3, 2022. Accessed December 12, 2022. https://www.fda.gov/news-events/fda-voices/how-fda-regulating-e-cigarettes

7. US Food and Drug Administration (FDA). Newly Signed Legislation Raises Federal Minimum Age of Sale of Tobacco Products to 21. Published October 2, 2022. Accessed December 12, 2022. https://www.fda.gov/tobacco-products/ctp-newsroom/newly-signed-legislation-raises-federal-minimum-age-sale-tobacco-products-21

8. Nguyen HV. Association of Canada’s Provincial Bans on Electronic Cigarette Sales to Minors with Electronic Cigarette Use among Youths. *JAMA Pediatrics*. 2020;174(1). doi:10.1001/jamapediatrics.2019.3912

9. Health Canada. Vaping product regulations. Published May 17, 2021. Accessed December 12, 2022. https://www.canada.ca/en/health-canada/services/smoking-tobacco/vaping/product-safety-regulation.html

10. UK Public General Acts. Children and Families Act 2014. Published 2014. Accessed December 12, 2022. https://www.legislation.gov.uk/ukpga/2014/6/contents/enacted

11. New South Wales (NSW) Government. E-cigarettes - Tobacco and smoking. Published 2021. Accessed December 12, 2022. https://www.health.nsw.gov.au/tobacco/Pages/e-cigarettes.aspx

12. Australian Government (Department of Health and Aged Care). About e-cigarettes. Published 2021. Accessed December 12, 2022. https://www.health.gov.au/topics/smoking-and-tobacco/about-smoking-and-tobacco/about-e-cigarettes
