# Supplementary material for: How do underage youth access e-cigarettes in settings with minimum age sales restriction laws? A scoping review
Source: BMC Public Health. 2023 Sep 18;23:1809. doi: 10.1186/s12889-023-16755-9 (PMC10506222; doi:10.1186/s12889-023-16755-9)
Supplement: Supplementary file 3 — Additional file 3. Detailed characteristics of included studies. [file 12889_2023_16755_MOESM3_ESM.docx]

Additional File 3: Detailed Characteristics of Included Studies

Table S3: Key characteristics and data variables from 17 studies Included in Scoping Review (Surveys, Focus Groups and Interviews)

| Citation | Setting | Study Type | Methods | # Part. | Age | EC use status | Implementation Date: Min. Age Sales Law(s) | Data Collection | Access Parameter(s) Reported | Conflicts of Interest | Funding Sources Noted |
| --- | --- | --- | --- | --- | --- | --- | --- | --- | --- | --- | --- |
| Alexander et al., 2018: *Youth who use e-cigarettes regularly: A qualitative study of behavior, attitudes, and familial norms* | USA | Focus groups | 4 focus group discussions in North Carolina (NC) and Florida; semi-structured | 41 | Mean=  15.8 y  Range=  14-17 y | Regular EC users (some or every day in past 30) | Aug. 2013: sales to <18 prohibited in NC; Jul 2014: sales to <18 prohibited in Florida | 2016  (Jan.-Feb.) | EC product use initiation; purchase location (qual.) | Nil | Nothing noted |
| Baker et al., 2019: *Youth source of acquisition for E-Cigarette* | USA | Survey | Data acquired from North Carolina Youth Tobacco Survey; paper-based delivery in schools | 640 | Range=  9-17 y | Current (past 30-d) EC users | Aug. 2013: sales to <18 prohibited in North Carolina | 2017  (exact time frame unclear) | Main product sources among current EC users | Nil | Cooperative agreement no. DP005974, funded by Centres for Disease Control and Prevention (CDC) |
| Braak et al., 2020: *How are adolescents getting their vaping products? Findings from the international tobacco control (ITC) youth tobacco and vaping survey* | USA, Canada, England | Survey | Data acquired from International Tobacco Control Policy Evaluation Project Youth Tobacco and Vaping Survey; online survey delivery | 2,779 | Range=  16-19 y | Current (past 30-d) EC users | USA: Aug. 2016, sales to <18y prohibited federally; Canada: bans to <18 or <19 by Sept. 2016 in 7 of 10 provinces;  England: Mar. 2014, federal sales ban to <18 | 2017  (Jul.-Aug.) | Main product source among current EC users | Nil | Grants from National Cancer Institute and Canadian Institute of Health-Public Health Agency of Canada; support from Hollings Cancer Centre, Univ. of South Carolina |
| Cwalina et al., 2021: *Prevalence of Young Adult Vaping, Substance Vaped, and Purchase Location across Five Categories of Vaping Devices* | USA | Survey | Data acquired from Wave 9 of Happiness and Health Study; online survey delivery | 1,174 | Mean=  19.2 y  Range=  18-20 y | EC ever-users | June 2016: California implemented T21 law, raising min. legal purchase age to 21 | 2018-2019  (Jun.-Oct.) | Main product sources among EC ever-users | Nil | Grants from National Cancer Institute at the NIH; FDA Centre for Tobacco Products; National Institute for Drug Abuse; Tobacco Related Disease Research Program |
| Groom et al., 2021: *The Influence of Friends on Teen Vaping: A Mixed-Methods Approach* | USA | Survey | Mixed-methods approach including online survey delivery | 1,549 | Range=  13-18 y | EC ever-users | Aug. 2016, sales to <18y prohibited federally; federal T21 law had not yet been implemented | 2017  (Aug.-Oct.) | First product source among EC ever-users | Nil | Grants from the National Heart, Lung, and Blood Institute of the NIH; FDA Centre for Tobacco Products |
| Mantey et al., 2019:  *Retail Access to E-cigarettes and Frequency of E-cigarette Use in High School Students* | USA | Survey | Data acquired from Youth Risk Behaviour Surveillance System | 1,217 | Range=  14-17 y | Current (past 30-d) EC users | Aug. 2016, sales to <18y prohibited federally; federal T21 law had not yet been implemented | 2017  (exact time frame unclear) | Main product source among current EC users | Nil | Nothing noted |
| McKeganey et al., 2019: *Sources of youth access to JUUL vaping products in the United States* | USA | Survey | Cross-sectional, online survey delivery | 1,537 | Range=  13-17 y | Current (past 30-d) EC users | Aug. 2016, sales to <18y prohibited federally; federal T21 law had not yet been implemented | 2018  (Nov.-Dec.) | Main source of JUUL among current EC users | Centre for Substance Use Research received funding from JUUL Labs Inc. to undertake research on the impact of its vape products | Funding provided by JUUL Labs Inc. (authors state funder had no input or control over study design, survey, sample recruitment, data analysis, interpret., or reporting of findings) |
| Merianos et al., 2019: *Characteristics of Daily E-Cigarette Use and Acquisition Means Among a National Sample of Adolescents* | USA | Survey | Data acquired from 2016 National Youth Tobacco Survey; paper-based delivery in schools | 1,579 | 23.1% middle school; 76.9% high school; mean and range not provided | Current (past 30-d) EC users | Aug. 2016, sales to <18y prohibited federally; federal T21 law had not yet been implemented. *At time of survey, sales to <18 were already prohibited in 47 US states and two territories | 2016  (Feb.-Jun.) | Main product sources among current EC users | Nil | Grants from National Institute on Drug Abuse (NIH), National Institute of Environmental Health Sciences (NIH); Medical Research Institute/ AAP Julius B Richmond Centre of Excellence |
| Meyers et al., 2017: *Access to Tobacco Among California High School Students: The Role of Family Members, Peers, and Retail Venues* | USA | Survey | Paper-based survey delivery in schools | 499 | Mean= 16.13 y | EC ever-users | Sept 2010: California implemented law restricting sales to (<18); California T21 law not yet been implemented | 2014-2015 (exact time frame unclear) | Most recent product source among EC ever-users; purchase location among EC ever-users | Nil | Grant from the National Cancer Institute; FDA Centre for Tobacco products |
| Nguyen, 2020: *Association of Canada's Provincial Bans on Electronic Cigarette Sales to Minors with Electronic Cigarette Use Among Youths* | Canada | Survey | Data acquired from Canadian Tobacco, Alcohol and Drugs Survey-CTADS | 8,212 | Mean= 16.4 y | Current (past 30-d) EC users | Provincial bans on sales to youth <18 or 19y (depending on province) implemented between 2015 and 2017;  followed by 2018 nation-wide ban on sales to <18 | 2017  (exact time frame unclear) | Use of social sources among current EC users | Nil | Grants from Canada Research Chair in Health Policy Evaluation; Canadian Institutes of Health Research; Janeway Children’s Hospital Foundation |
| Pepper et al., 2019: *How Do Adolescents Get Their E-Cigarettes and Other Electronic Vaping Devices?* | USA | Survey | Cross-sectional, online survey delivery | 1,729 | Mean=  16.1 y; Range=  15-17 y | Current (past 30-d) EC users | Aug. 2016, sales to <18y prohibited federally; federal T21 law had not yet been implemented | 2016/2017  (exact time frame unclear) | Main source of primary device among current EC users | Nil | Internal funds from RTI (independent, non-profit US research institute); RTI noted to have no other role in study |
| Schiff et al., 2020: *Accessing Vaping Products When Underage: A Qualitative Study of Young Adults in Southern California* | USA | Interviews | In-depth structured interviews (30-90 mins); participants reflected back on experiences when under legal age | 61 | Mean=  20.9 y  Range=  18-25 y | Current (past 30-d) EC users | Sept 2010: California implemented law restricting sales to <18;  2016: California implemented state-wide T21 law | 2018-2019  (Jun.-Jun.) | EC product use initiation; source/ location (qual.); device sharing (qual.);  purchase success (qual.) | Nil | Grants from National Institute on Drug Abuse (NIH) and National Cancer Institute at (NIH) |
| Schiff et al., 2021: *E-cigarette and cigarette purchasing among young adults before and after implementation of California's tobacco 21 policy* | USA | Survey | Data acquired from Southern California Children's Health Study; online survey delivery | 1,310 | Mean=  20.2 y | Current (past 30-d) EC users | Sept 2010: California implemented law restricting sales to <18;  June 2016: California implemented state-wide T21 law | 2016-2017  (Oct.-Oct.) | Main product source among current EC users | Nil | Grants from National Cancer Institute (NIH) Centre for Tobacco Products (FDA); National Institute for Drug Abuse (NIH); Tobacco Related Disease Research Program |
| Tanski et al., 2019: *Youth Access to Tobacco Products in the United States: Findings From Wave 1 (2013–2014) of the Population Assessment of Tobacco and Health Study* | USA | Survey | Data acquired from Wave 1, Population Assessment of Tobacco and Health (PATH) Study; longitudinal cohort | 6,553 | Range=  15-17 y | Current (past 30-d) EC users | At time of survey, EC sales to <18 were already prohibited in 47 US states and two territories (may not have been robust enforcement due to limited resources; federal laws banning sales to <18y not yet implemented | 2013-2014  (Sept.-Dec.) | Main product source among current EC users | Nil | Federal funds received from the National Institute on Drug Abuse (NIH) and FDA, Department of Health and Human Services, under a contract to *Westat* [a US-based professional services company] |
| Wagoner et al., 2021: *Adolescent Use and Perceptions of JUUL and Other Pod-Style e-Cigarettes: A Qualitative Study to Inform Prevention* | USA | Focus groups | Six focus group discussions (60-90 min.) in North Carolina (NC); facilitated using semi-structured interview guide | 29 | Mean=  15.9 y | EC ever-users | Aug. 2013: NC State implemented law restricting sales to <18; federal T21 law not yet implemented at time of study | 2019  (Jun.) | EC product use initiation; source (qual.);  Device sharing behaviour (qual.) | Nil | Grant from National Cancer Institute issued to the Wake Forest Baptist Comprehensive Cancer Centre |
| Wang et al., 2021: *Characteristics of e-Cigarette Use Behaviors Among US Youth, 2020* | USA | Survey | Data acquired from National Youth Tobacco Survey; school-based self-administered survey | 1,764 | Mean and range not provided, gr. 6-12 | Current (past 30-d) EC users | Dec. 2019: federal T21 law raised minimum age of sale of tobacco products (incl. ECs) from 18 to 21 | 2020  (Jan.-Mar.) | Main product sources among current EC users | Nil | Funding from Centres for Disease Control and Prevention (CDC); FDA |
| Watts et al., 2022: *Vaping product access and use among 14–17-yearolds in New South Wales: a cross-sectional study* | Aus. | Survey | Data acquired from Generation Vape study; online cross-sectional survey | 233 | Range=  14-17 y | EC ever-users | Dec. 2015: sale to minors <18 prohibited in NSW; national law requiring a prescription to legally access nicotine-containing EC products had not yet been implemented | 2021  (Sept.) | Most recent product source among EC ever-users | Nil | Funding from NSW Ministry of Health and the Minderoo Foundation [an Australian philanthropic organisation] |
